# Plasmacytoid Dendritic Cells as a Novel Cell-Based Cancer Immunotherapy

**DOI:** 10.3390/ijms231911397

**Published:** 2022-09-27

**Authors:** Sabina Sánchez Hernández, Martin Roelsgaard Jakobsen, Rasmus O. Bak

**Affiliations:** Department of Biomedicine, Aarhus University, Høegh-Guldbergsgade 10, 8000 Aarhus, Denmark

**Keywords:** plasmacytoid dendritic cells, pDC-based vaccines, tumor-specific immune responses, melanoma

## Abstract

Plasmacytoid dendritic cells (pDCs) are multifaceted immune cells with a wide range of innate and adaptive immunological functions. They constitute the first line of defence against multiple viral infections and have also been reported to actively participate in antitumor immune responses. The clinical implication of the presence of pDCs in the tumor microenvironment (TME) is still ambiguous, but it is clear that pDCs possess the ability to modulate tumor-specific T cell responses and direct cytotoxic functions. Therapeutic strategies designed to exploit these qualities of pDCs to boost tumor-specific immune responses could represent an attractive alternative compared to conventional therapeutic approaches in the future, and promising antitumor effects have already been reported in phase I/II clinical trials. Here, we review the many roles of pDCs in cancer and present current advances in developing pDC-based immunotherapeutic approaches for treating cancer.

## 1. Introduction

Switching on effector T cell responses against tumor-associated antigens (TAAs) while maintaining the immune homeostasis is essential for the immune system to fight cancer. Immune responses against tumors rely mainly on effector CD8+ T cells, also known as CD8+ cytotoxic T lymphocytes (CTLs), and the presentation of TAAs by antigen-presenting cells (APCs). This constitutes the first steps of the cancer-immunity cycle [1]. The major group of specialized APCs involved in effector T-cell priming includes Dendritic cells (DCs), which process and present TAAs on major histocompatibility complex (MHC) molecules acquired from the tumor microenvironment (TME) [2]. Many new immunotherapy strategies seek to boost antitumor T cell responses to dispose the tumor efficiently. In this respect, DC-based immunotherapy constitutes an exciting field to explore with their multifaceted roles in supporting antitumor responses, including antigen-presenting capacity, DCs migratory properties, and their role in cytokine production. DCs obtained from patients can be stimulated and loaded with antigens of interest in vitro and reinfused into patients to induce antigen-specific T cell responses. Thus, it has been a goal of cancer immunotherapy to develop customized DC-based vaccines that are effective and safe, a strategy that could also be applied for viral diseases [3].

Based on their source, phenotype, and function, DCs comprise various subsets of cells that can be binned into the two major subsets of conventional DCs (cDCs) and plasmacytoid DCs (pDCs). Two major lineages have long been distinguished in the cDC subset: CD141+ cDC1 and CD1c+ cDC2 [4]. However, a more detailed picture of the complexity of DCs is gradually emerging. At least six clusters of DCs and four of monocytes were recently identified by single-cell RNA profiling of peripheral blood mononuclear cells (PBMCs). Within the identified groups of DCs, a new subpopulation emerged with a transcriptional profile resembling that of a pDC signature. This new cluster represents around 2–3% of blood DCs and express unique markers, including AXL, a receptor tyrosine kinase implicated in several physiological processes and the transmembrane receptor SIGLEC6, which binds to sialic acids [5]. The subset was, therefore, termed AXL + SIGLEC6+ DCs (AS DCs). AS DCs were, however, found to be functionally distinct from pDCs and did not express genes associated with traditional pDC functions such as pathogen sensing and the expression and secretion of type I IFNs [5]. Although new technologies for transcriptome profiling are enabling researchers in this field to characterize novel clusters within the DC lineage, their heedless categorization can sometimes lead to confusion. One challenge is distinguishing cell subsets from cell states when proposing reannotation of DC subset nomenclatures, as Ginhoux et al. pointed out in a recent publication [6].

Until now, most DC-based vaccination strategies that have been explored are based on monocyte-derived DCs (moDCs) and cDC2s, with only a few clinical trials evaluating pDC-based vaccines. Here, we will focus on the much less studied and enigmatic pDCs, their natural functions and characteristics, identified roles in cancer, and their potential as a novel anticancer immunotherapy.

## 2. pDC Biology: Phenotype and Function

Firstly observed in 1958 in the T cell zones of human lymph nodes [7], a unique subset of cells was identified. They were initially called plasmacytoid T cells or plasmacytoid monocytes based on their plasma cell-like morphology and shared markers with T cells and monocytes. However, decades later, these cells were discovered to produce large amounts of type I Interferon (IFN-I) in response to viral infections and were then re-named natural interferon-producing cells. In the late 1990s, they were once more renamed to reflect some of their obvious similarities with conventional DCs in many aspects of innate and adaptive immune functions [8,9].

pDCs are generated in the bone marrow (BM) and recent data suggest that they mainly derive from lymphoid progenitors [10,11,12]. They are mainly found in lymphoid organs and peripheral blood, where they represent less than 1% of the mononuclear cellular pool. The differentiation of pDCs from hematopoietic stem and progenitor cells requires Fms-like tyrosine kinase 3-ligand (Flt3L) [13] and Interleukin 3 (IL-3) [14,15], among other cytokines. Through endosomal Toll-like receptors (TLRs) 7 and 9, mature pDCs recognize viral nucleic acids and bacterial CpG DNA and activate the IFN-I signaling pathways. IFN I induction by TLR7 and TLR9 ligands depends on interactions between the adaptor molecule MyD88 and Interferon Regulatory Factor 7 (IRF7) [16]. It has been shown at single-cell levels that only a small fraction of pDCs produces IFNα, and although TLR signaling is necessary for induction of type I IFN, it is not sufficient. The microenvironment considerably influences Type I IFN responses, and an autocrine loop via the type I IFN receptor (IFNAR) enhances TLR-induced IFNα production [17].

Interestingly, a broad immune response can be observed after pDC activation, which includes the secretion of multiple chemokines and cytokines such as (i) TNF-α, IL-6, CCL2, CCL3, CCL4, CXCL8, CXCL9, CXCL10, and CXCL11; (ii) the expression of migratory receptors such as CCR2, CCR5, CCR6, CCR7, CXCR4, CD62L, PSGL1, and β1/β2 integrins; and (iii) the elevated expression of MHC class I and II and costimulatory molecules CD80, CD83, and CD86 on their surface [18,19,20,21].

Human pDCs have been described as negative for lineage markers (CD3, CD14, CD16, CD19, CD20, and CD56) and CD11c and positive for CD123 (IL3Rα), CD303 (CLEC4C /BDCA2), CD304 (NRP1 /BDCA4), and HLA-DR [22]. Nonetheless, rather than a defined subset of DCs, pDCs are phenotypically heterogeneous. For instance, based on the expression of CD2, two subsets of human blood pDCs with transcriptional and functional differences have been identified (CD2High and CD2Low pDCs) [23,24]. Compared to CD2Low pDCs, CD2High pDCs appear to produce lower amounts of IFNα but have superior resistance to stress-induced apoptosis and a higher ability to migrate and stimulate T cells [24]. Furthermore, three distinct pDC subpopulations (termed P1–P3) with specialized innate and adaptive functions were identified in a previous study showing the following clusters: PD-L1+CD80− (P1), PD-L1+CD80+ (P2), and PD-L1–CD80+ (P3) [25]. P1-pDCs (PD-L1+CD80−) specialized in type I IFN production, P3-pDCs (PD-L1–CD80+) contributed to T cell activation through antigen presentation, and P2-pDCs (PD-L1+CD80+) contain both innate and adaptive functions [25]. The identification of pDCs based on the expression of surface markers is further complicated by the discovery of the aforementioned AS DCs that have some phenotypic similarities to pDCs (e.g., expression of CD123, CD304, IGJ, and MZB1) [4,5,25]. Nonetheless, AS DCs can be distinguished by their expression of unique markers absent in pDCs (e.g., CD33, CD5, and AXL) [5,25]. Finally, a very recent single-cell RNA sequencing study revealed the existence of not less than 9 subclusters in unstimulated pDCs [26]. Unfortunately, it was not possible to identify cell surface markers that could discriminate these clusters. Upon stimulation with influenza virus, this study elegantly demonstrated that a minor single cluster of pDCs mainly produces the totality of IFN-I, IFN-III, and the majority of induced cytokines in line with previous observations [26].

The pDC specification program in hematopoietic progenitors has been reported to begin with IRF8 and requires the expression of transcription factors TCF4 (E2-2), BCL11A, and SPIB. On the contrary, ID2 impairs the development of pDCs and enhances cDC1 generation [27]. At a single cell level, the loss of TCF4 and gain of ID2 have been observed in one cluster of activated pDCs, thereby indicating that pDCs might adopt some plasticity towards a cDC/APC-like phenotype [26].

## 3. pDCs in Cancer

Our immune system is in charge of detecting and eliminating tumor cells while preventing tumor growth—an essential function known as immune surveillance. Tumor cell killing relies mainly on tumor-specific CTLs, after the cross-presentation of TAAs by DCs to naive T cells in lymph nodes [1]. Additionally, CD4+ T cells can reinforce CTL-mediated antitumor responses by producing IFN-γ, which increases MHC class I antigen processing and presentation [28]. Other immune cells involved in antitumor immune responses include natural killer (NK) cells and macrophages. However, immune responses to cancer frequently fail to completely eradicate tumor cells and inhibit tumor progression effectively. Different cancer therapies aim to overcome this issue and stimulate tumor-specific immune responses. With the same purpose, the idea of exploiting the unique immune properties of pDCs to treat cancer is being investigated in some solid tumors [29,30,31].

**Figure 1:**
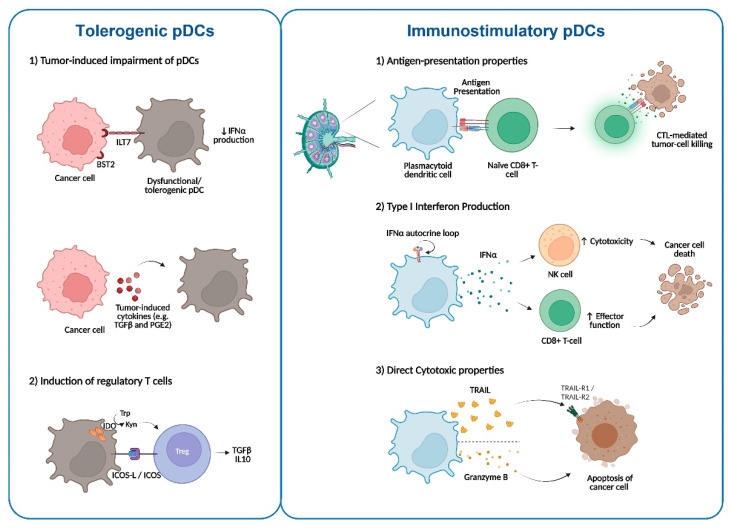
Plasmacytoid dendritic cells play a dual regulatory role in cancer immune responses. pDCs that exhibit a tolerogenic phenotype (**left panel**) contribute to the tumor immune escape. At the same time, signals released from tumor cells and the TME (e.g., the expression of BST2 in cancer cells or tumor-derived cytokines) favor the impairment of pDCs. On the other hand, functional pDCs (**right panel**) can trigger anti-tumor immune responses through their antigen-presenting capacity and cytotoxic functions.

Despite the potential of pDCs to trigger antitumor immune responses, the infiltration of pDCs in the TME has been associated with both improved and reduced prognosis in different types of cancers, including head and neck cancer [32,33], breast cancer [34,35,36,37], melanoma [38,39], pancreatic ductal adenocarcinoma [40], colon cancer [41], and lung cancer [42]. Hence, the specific microenvironmental context seems to determine whether pDCs will display active immunity functions or be subject to or involved in immune tolerance (Figure 1).

As an example of pDCs associated with improved outcomes in cancer patients, an immunostimulatory pDC subset that expresses high levels of OX40 (OX40+ pDCs) and lacks ICOS-L expression was recently identified in the TME of head and neck squamous cell carcinomas (HNSCC), especially in patients with HPV+ HNSCC [33]. OX40+ pDCs presented a more mature and activated phenotype based on an increased expression of various maturation markers (CD40, CD80, CD86, OX40L, Siglec6, and Axl) and the elevated production of TRAIL, Granzyme B, and IFN-α upon activation. Transcriptomic analyses showed significantly higher levels of the pDC-defining genes IRF8 and E2-2 (TCF4) in OX40+ pDCs and the preferential expression of antigen presentation-related genes (CD40, CD80, CD86, OX40L, and TLR signaling). The presence of OX40+ pDCs in the TME positively correlated with survival in HNSCC patients [33]. Furthermore, enrichments in CD8+ T cells, pDCs, and PDL1High ICOSLow cDC2s with a secretory profile correlated with a high infiltration of CD3+ T cells and good prognosis in HNSCC patients [43].

The migration of pDCs to the TME could be directed through the CCL20/CCR6 axis, as proposed in melanoma patients [38]. While circulating pDCs from healthy individuals expressed low levels of the chemokine receptor CCR6, the upregulation of its expression was observed in 36% of melanoma patients included in this study. The ability of CCR6-expressing pDCs to migrate in response to CCL20 was demonstrated in transwell migration assays, suggesting that CCL20 produced in the TME might participate in recruiting CCR6-expressing pDCs in melanoma tumors [38]. Hypoxic conditions in the TME could also promote the recruitment of pDCs in the tumor. This has been observed in hepatocellular carcinoma where a hypoxic TME induces adenosine production and its extracellular accumulation, which facilitates the tumor-infiltration of pDCs via the adenosine A1 receptor expressed in circulating pDCs [44].

### 3.1. Impairment of pDCs in the TME

Several mechanisms have been described to be causative for the impairment of pDCs by the immunosuppressive TME. One of those mechanisms is speculated to be the tumor cell secretion of a pool of cytokines that can stimulate a tolerogenic state in pDCs. As an example, tumor-derived prostaglandin E2 (PGE2) and transforming growth factor- β (TGF- β) synergistically suppress the production of IFN-I and tumor necrosis factor (TNF) in activated pDCs, which then exhibit a tolerogenic phenotype [45]. Tumor cells and cells in the TME (e.g., myeloid-derived suppressor cells, tumor-associated macrophages, γδ T cells, and NK cells) could impair pDC function through different mechanisms [46]. For example, the overexpression of bone marrow stromal antigen 2 (BST2), also known as ILT7 ligand or tetherin, has been described on the surface of various human cancer cells. Thus far, the best characterized role of BST2 is to limit the release of enveloped viruses from infected cells during viral infections [47]. BST2 is an IRF-1-inducible gene that binds to the immunoglobulin-like cell transcript 7 (ILT7), an inhibitory receptor predominantly expressed by pDCs [48]. It has been proposed that IFN-α secreted by pDCs may induce BST2 on stromal cells in the TME, which would result in the suppression of IFN-α production as a consequence of a negative feedback loop [49].

The accumulation of regulatory T cells (Tregs) with strong immunosuppressive capacity has also been associated with poor prognosis in certain cancers [50]. It has been observed that activated pDCs can promote the differentiation of CD4+CD25− T cells to CD4+ CD25+ Foxp3+ Tregs, with immunosuppressive effects on naive CD4+ T cell proliferation and a cytokine profile characterized by the enhanced production of IL-10, TGF-β, IFN-γ and IL-6 [51]. The immunosuppressive phenotype of Tregs is regulated by the immunoregulatory enzyme indoleamine 2,3-dioxygenase (IDO), an enzyme that participates in tryptophan catabolism and is expressed by pDCs [52,53]. Moreover, it has been proposed that IDO maintains the suppressive phenotype of Tregs by blocking IL6 production in activated pDCs [53]. A relatively rare subpopulation of CD2Hi, CD5+, and CD81+ pDCs can trigger Treg differentiation more efficiently than CD2Hi CD5− CD81− pDCs, as has been observed in co-cultures of each pDC subset with naïve CD4+ T cells [54]. CD2Hi, CD5+, and CD81+ pDCs have been found in human peripheral blood and also in the bone marrow and cord blood, and the production of IDO is also slightly superior in this subset of pDCs compare with CD2Hi CD5− CD81− pDCs. At the same time, CD2Hi CD5+ CD81+ pDCs display a reduced capacity in producing IFNα [54]. It has also been described that the expansion and function of Foxp3+ ICOS+ Treg cells depend on ICOS costimulation provided by tumor-infiltrating pDCs expressing high levels of ICOS-ligand (ICOS-L) [55,56].

One aspect that would be relevant to explore is to what extent the tolerogenic phenotype of tumor-associated pDCs can be reversed. For instance, the inhibition of the ubiquitin receptor Rpn13 reverts the immunosuppressive phenotype of Multiple Myeloma-associated pDCs and restores pDC-induced T cell cytolytic activity against tumor cells [57], although the process that enables this change remains unclear. In addition, PGE2 inhibitors and IDO inhibitors are being used in preclinical and clinical settings to restrain immunosuppressive circuits that drive tumor immune evasion and restore an immunostimulatory TME [58]. Using in vitro and in vivo models, it has been shown that IDO inhibitors allow the conversion of Tregs to Th17-like T cells in a reprogramming process that requires activated CTLs and IL-6 produced by activated pDCs [53]. Concurrently, IDO-inhibitors rescued the production of IL-6 by pDCs, which IDO suppresses through the upregulation of an inhibitory isoform of the transcription factor NF-IL-6, which is essential for IL-6 gene transcription [53].

### 3.2. pDCs in Antitumor Immunity

pDCs have the capacity to mediate innate and adaptive immune responses against tumor cells. Through the secretion of the cytolytic molecules such as Granzyme B and TRAIL, pDCs have been shown to display direct cytotoxic properties against tumor cells. The low levels of Granzyme B and TRAIL produced by steady-state pDCs increase considerably after activation, and it has been proven that TLR-activated pDCs have the ability to kill several tumor-derived cell lines, including hematological cancer cells, breast cancer cells, and melanoma cells [23,59,60].

Antitumor effects of IFNs—of which pDCs are the most productive cell type in the body—have also been widely described, and it is known that IFNs hamper tumor progression by regulating different physiological processes such as angiogenesis. IFNs induce the transcription of a few hundred genes, known as interferon-stimulated genes (ISGs), establishing immunoregulatory and antitumor networks. Thereby, IFNs restrain tumor cell growth and migration and can activate TAA presentation. Furthermore, type I IFNs activate NK cells and CD8+ T cells [61]. Recombinant IFNα2 was the first cancer immunotherapy approved by the US Food and Drug Administration (FDA) for melanoma treatment, and the administration of IFNα and IFNβ improved patient outcomes with different malignancies. However, extending the therapeutic use of IFNs was challenging due to their short half-life and systemic side effects often related to IFN therapies [61,62].

Another antitumor feature of pDCs is the uptake and transport of antigens from peripheral tissues to lymphoid organs where the MCH-I antigen’s cross-presentation to CD8+ T cells can take place [63]. TAAs derived from proto-oncogenes and genes overexpressed in the tumor microenvironment can be captured by tumor-infiltrated APCs (including pDCs), which transport them to the lymph nodes and process them into peptides that will be presented through MHC molecules to naïve T cells, thus initiating antitumor immune responses. C-type lectin-like receptors (CLR) constitute a group of cell surface receptors that promote the endocytosis of antigens into APCs. On their surface, human pDCs express different receptors involved in antigen capture and presentation, including DCIR, BDCA-2 (CD303), and BDCA-4 (CD304) [22,64]. Ly75, also known as DEC-205, is a CLR expressed by different immune cells, including pDCs, where a role in antigen uptake and presentation in a clathrin-dependent manner has been demonstrated [65]. Interestingly, contrary to most endocytic receptors that are downregulated in DCs upon activation, Ly75 is overexpressed in mature pDCs and moDCs [65]. Given this, vaccines based on pDCs loaded with TAAs could have the potential to boost tumor-specific T cell responses.

In addition, TNF-α secreted by pDCs promotes antigen processing and presentation and enhances T cell activation but seems to negatively regulate other functions of pDCs, including their central role as IFN-α-producing cells [66]. RNA sequencing data revealed transcriptional changes in blood-purified human pDCs treated with exogenous TNFα. TFNα-treated pDCs lost their main role as IFNα-producing cells and acquired a phenotype closer to cDCs. TNFα promotes the upregulation of genes associated with MHC-I and MHC-II Ag processing and presentation; costimulatory molecules and chemokine receptors, including CD80, CD86, HLA-DR, and CCR7; and genes associated with T cell differentiation, while negatively regulating the expression of genes involved in the TLR cascade signalling and type I IFN secretion [66]. Again, this highlights the potential plasticity of pDC phenotype and function.

## 4. pDC-Based Cancer Immunotherapy

The unique immunostimulatory properties of pDCs can be exploited in cancer immunotherapy, either through treatments destined to enhance these qualities of existing pDCs directly in vivo or by utilizing pDCs as a cell therapy for adoptive transplantation. In the first case, targeting TLR signaling pathways is the main therapeutic strategy that has been employed to activate pDCs in vivo. On the other hand, the development of novel pDC-based vaccines for treating tumors and infectious diseases is the goal for cell therapies in exploiting the immune properties of pDCs, with the potential of endowing pDCs with new or enhanced properties by genetic engineering and synthetic biology.

### 4.1. Immunotherapy Strategies Based on Activation of TLR Signalling

The efficacy of various anticancer therapies can be increased by promoting TLR signaling, which in turn favors the activation of various immune cells, including NK cells and DCs. Several TLR agonists have proven their potential for cancer treatment in different experimental and clinical settings [67]. The antitumor properties of TLR7, TLR7/8, and TLR9 agonists are mainly mediated by the activation of pDCs. In fact, it has been shown in stage I/III melanoma patients that the local administration of CpG-B (also called CpG 7909; PF-3512676), a TLR9 agonist, enhanced the activation state of pDCs and melanoma-specific CTLs responses and reduced Treg frequencies [68,69]. Along the same line, the increased expression of the costimulatory molecules CD83 and CD40 has been documented in pDCs from the sentinel lymph node (SLN) of stage I/II melanoma patients treated with CpG-B alone or in combination with GM-CSF [70]. Imiquimod (IMQ), a synthetic TLR7/8 agonist commonly used to treat epithelial skin tumors, activates TRAIL-mediated pDC cytotoxicity that in turn depends on IFN-α. Thus, human blood-derived pDCs previously stimulated with IMQ can effectively lyse melanoma cell lines that express TRAIL receptor-2 (TRAIL-R2) [71]. Although physiological levels of IFN-α only induce TRAIL to a limited extent, even suboptimal doses of IMQ could increase those levels [71]. The antitumor effects of IMQ and CpG connected to pDC activation have also been described in breast cancer models. Murine pDCs activated with IMQ or CpG express and release TRAIL and Granzyme B, and they elicit antitumor responses against breast cancer in vitro and in vivo [72]. In their study, Wu et al. observed more robust antitumor responses and superior survival in mice treated with IMQ-activated pDCs compared to CpG-activated pDCs.

### 4.2. DC Mobilization for Cancer Immunotherapy

Flt3L efficiently mobilizes DCs, including pDCs, into the peripheral blood and can be used as a vaccine adjuvant [73]. Under the hypothesis that Flt3L potentiates DC-mediated antigen presentation, pre-treatments with recombinant human Flt3L (CDX-301) before vaccination with CDX-1401, a DC-based vaccine targeting the tumor antigen NY-ESO-1, was tested in melanoma patients in a phase II clinical trial [74]. Antigen-specific T cell responses were reinforced in patients who received CDX-301. Furthermore, DC mobilization was observed in CDX-301-treated patients, where mean cDCs numbers in PBMCs increased 27.8-fold while pDCs numbers increased 15-fold [74]. Although this trial did not address roles of each DC subset in priming antitumor responses against the NY-ESO-1 antigen, the authors propose that they probably play distinct and complementary roles.

### 4.3. pDC-Based Vaccines for Cancer Immunotherapy

Cancer immunotherapies exploiting the antigen-presenting capacity of DCs have shown low efficacies in numerous clinical trials, mainly based on moDCs and cDC2s. This could, at least in part, be due to immunosuppressive signals displayed by tumor cells and within the TME. The tolerogenicity and dysfunction of DCs in the TME reduced the efficacy of DC vaccines. Moreover, most patients enrolled in the first clinical trials that evaluated DC-based vaccines were in the last stages of the disease [75]. Thus far, Sipuleucel-T (commercialized as Provenge) is the only DC-based vaccine approved by the FDA for metastatic castrate-resistant prostate cancer (CRPC). It is an autologous PBMC cell product that is activated with a prostate antigen fused to granulocyte-macrophage colony-stimulating factor (GM-CSF) to promote APC maturation [76]. It not only mainly consists of monocyte-derived APCs and DCs but also contains some T cells, B cells and Natural Killer (NK). One cell therapy batch consists of a minimum of 50 million CD54+ cells, and three separate batches were manufactured for three treatments.

New attempts that combine DC-based vaccines with other immunotherapies or traditional anticancer treatments are underway and early data seem promising, and great efforts are being made to increase their effectiveness [75]. However, the unique properties of pDCs render them an attractive alternative to traditional DC-based vaccine platforms that have been proven to be safe in first-in-human trials (Table 1). Current cancer immunotherapy trials evaluating pDC-based vaccines have drawn on pDCs from two sources: autologous pDCs obtained from peripheral blood and allogeneic pDC cell lines derived from leukemic pDCs [29,30,31]. Alternatively, we also suggest that HSPCs could represent an excellent source for the in vitro generation of pDCs that could be used in future clinical settings [77,78].

Cancer immunotherapy strategies based on pDC-vaccines presenting TAAs have primarily been evaluated in advanced melanoma patients. Since melanoma cells are highly immunogenic cells that express different TAAs such as Melan A, gp100, and tyrosinase [79], they are suitable for vaccine testing. The first clinical trial that tested the therapeutic potential of naturally circulating pDCs against malignant tumors was carried out in fifteen patients with metastatic melanoma expressing gp100 and tyrosinase (NCT01690377) [29]. In this trial, Tel et al. administered three doses of autologous pDCs purified from an apheresis product using the clinical-scale CliniMACS separation system and GMP-grade magnetic bead–coupled anti-BDCA4 antibodies. Twenty-four hours prior to infusion, pDCs were activated with FSME-IMMUN, an inactivated whole-virus vaccine that acts as a natural TLR agonist, and then loaded with melanoma-associated peptides. A maximum of three cycles of three biweekly injections were performed intranodally in the patients. Although six patients experienced grade 1 flu-like symptoms, the vaccines were well tolerated, without evidence of severe toxicity. Activated pDCs were highly mature, based on the expression levels of CD80, CD83, CD86, MHC class I, and class II, and they demonstrated their ability to migrate to distinct lymph nodes in vivo. Furthermore, evidence on tumor-specific CD4+ and CD8+ T-cell responses and the upregulation of the IFN signature was documented, even with small numbers of administered pDCs in the range of 0.3–3 million cells per dose [29]. Although no significant conclusions could be drawn about the clinical outcome due to the low patient number, comparisons to carefully selected and matched historical control patients receiving standard chemotherapy showed a superior median progression-free survival (PFS) in vaccinated patients (4 versus 2.1 months), and the overall survival (OS) was notably improved compared to matched control patients (22 versus 7.6 months). Additionally, 47% of the patients that received pDC vaccinations were alive even two years after the start of the clinical trial, compared with 8.33% of the patients treated with standard dacarbazine chemotherapy.

Despite the therapeutic potential demonstrated in this first clinical trial, the low frequency of circulating pDCs may represent a limiting factor for developing immunotherapeutic strategies based on autologous pDCs. To overcome this limitation, the ability to initiate protective immune responses of a human pDC line derived from HLA-A*0201 leukaemic pDCs has been evaluated first in vitro and in humanized mice and, finally, in melanoma patients [31,80,81]. Due to the use of transformed cancer pDCs, the cells are irradiated to prevent tumorigenesis. Antitumor responses triggered by this irradiated pDC line were confirmed in tumor-bearing humanized mice that received a subcutaneous injection of irradiated pDCs loaded with melanoma-derived antigens where CD8+ T-cells were present in the tumor site and the draining lymph nodes [80]. The amplification of central/effector memory tumor-specific CTLs was observed for peripheral blood mononuclear cells (PBMCs) and tumor-infiltrating lymphocytes (TILs) samples from stage I–IV HLA-A*0201 melanoma patients stimulated with pDCs loaded with peptides derived from four melanoma TAAs (MelA, gp100, tyrosinase, and MAGE-A3). The central/effector memory phenotype acquired by pDC-primed CTLs promoted long-term persistence and antitumor efficacy in the mice, and CTLs also demonstrated the ability to kill melanoma tumor cells in vitro [81]. The HLA-A*0201 pDC line was then evaluated for safety and capacity to trigger antitumor responses in metastatic melanoma patients in a phase I clinical trial (NCT01863108) [31]. Although the patient cohort was small (nine patients), three weekly injections of up to 60 × 10^6^ pDCs in stage IIIC/IV HLA-A*0201 melanoma patients were safe and well tolerated. No detectable allo-response to the vaccine was observed, even at the highest dose, and no patient stopped the treatment as a consequence of treatment-derived side effects. However, four patients with progressive disease were withdrawn from the trial to start a different therapeutic strategy. Two patients showed enrichment of antitumor memory T-cells in peripheral blood compared to baseline, and tumor-specific T cells were detected in the metastasis of one patient. However, anti-vaccine T cells from the metastasis expressed high levels of PD-1, and the PD-1/PD-L1 axis probably limited their antitumoral activities in the invasive margin, in which tumor cells and macrophages expressed PD-L1. A similar approach is being evaluated for treating HLA-A*02:01 lung cancer patients in a phase I/II trial (NCT03970746). Although this therapeutic strategy does not require the isolation of autologous pDCs and the number of pDCs is not a limiting factor, vaccination with this pDC line has been restricted to HLA-A*02:01 patients. In this regard, to improve this pDC line and to make it more widely available, its transduction with retroviral vectors encoding new HLA molecules has been tested recently [82]. Efficient transduction (~80%) was feasible without compromising functionality and cell state, and these transduced cells showed antigen-presentation capacities through acquired HLA molecules. The same strategy has been used to generate pDCs that endogenously express antigens of interest. Interestingly, pDCs expressing the antigens of interest by viral transduction were able to trigger the in vitro expansion and activation of antigen-specific T cells, although higher frequencies of specific T cells were observed using passively loaded pDCs compared to transduced pDCs [82].

The crosstalk that DCs establish with other immune cells can contribute greatly to the immune responsiveness of DC-based cancer vaccines, and the ability of DCs to attract and engage with other immune cell types is, therefore, paramount. The chemoattractive properties of pDC and cDC2-based vaccines have recently been evaluated by van Beek et al., both in vitro and in patient-derived skin biopsies from previous DC vaccination trials conducted in metastatic melanoma patients (NCT01690377) [20]. The authors found that both DC subsets exhibited a distinct expression pattern of chemokines connected to a different ability to recruit immune effector cells. Compared to cDC2, activated pDCs showed a greater expression of CXCL9, CXCL10, and CXCL11, which are all ligands for C-X-C motif chemokine receptor 3 (CXCR3), and pDCs increased the CXCR3-dependent recruitment of CD8+ T cells, CD56+ NK-like T cells and unconventional γδ T cells in migration assays using bulk PBMCs [20]. These immune cell populations were also found in cultures of skin-infiltrating lymphocytes obtained from 48 stage III melanoma patients who received a combined pDC and cDC2 vaccine, suggesting the presence of these immune cells in the original skin biopsies. Interestingly, when the authors compared biopsies from patients who received FSME-stimulated pDCs [29] with those from patients who received GM-CSF-stimulated cDC2s [83], a stronger infiltration of CD8+ T cells was detected in patients vaccinated with pDCs, but tumor antigen-specific CD8+ T cells were only found in patients who had received cDC2 [20]. Based on these results, the authors suggest a vaccine that combines the chemoattractive properties of pDCs with the superior T cell priming capacity of cDC2s—a strategy that they have already tested in prostate cancer patients (NCT02692976) and is being evaluated in metastatic endometrial cancer (NCT04212377).

The above-mentioned blood-derived cDC2 and pDC combination vaccine has been investigated in a phase IIa trial in chemotherapy-naïve patients with CRPC (NCT02692976) [30]. Twenty-one patients were randomly divided into three equal groups to receive cDC2, pDCs, or combined cDC2 and pDC vaccinations. cDC2 and pDCs loaded with HLA-A*0201-binding peptides derived from three TAAs (NY-ESO-1, MAGE-C2 and MUC1) were activated with premixed protamine HCl and gp100 mRNA for 6h. Patients received 1 to 3 cycles of three biweekly intranodal injections in a tumor-free lymph node, with a six-month interval between cycles. DC vaccines were well tolerated, and only low-grade toxicity, including flu-like symptoms, was reported in the trial participants. Functional antigen-specific T cells were detected in peripheral blood from 12 patients (57%) after vaccination, correlating with longer PFS and greater IFNγ production. The median PFS in patients with TAA-specific T cells whose tumor expressed the same TAA was 10.7 months, compared to 5.2 months in patients that did not show this match. Interestingly, no significant differences in TAA-specific responses were observed between DC subsets [30]. In order to follow up on the quality of life of prostate cancer patients who received DC vaccines, patients completed a set of questionnaires assessing different health-related aspects (including physical and emotional aspects) at baseline and at different times after treatments. High health-related quality of life (HRQoL) was observed in patients who participated in this trial, with no evidence of deterioration during the vaccination period [84]. High HRQoL scores were observed up to one and two years after study enrolment in patients eligible for a second (13 patients) and third (7 patients) vaccination cycle [84]. This therapeutic strategy combining cDC2 and pDCs is also being evaluated in women with metastatic endometrial cancer (NCT04212377). However, to our knowledge, the reports have not been released yet.

## 5. Conclusions

Through their antigen-presenting capacity and direct cytotoxic functions, pDCs can participate in antitumor immune responses, and cancer immunotherapies exploiting those immunostimulatory properties are gradually emerging. Different therapeutic approaches both seek to enhance these qualities of pDCs in vivo or to develop cell therapy products based on pDCs. In the first case, the administration of TLR agonists has shown antitumor immune responses and clinical benefits in some tumors, linked to the activation of pDCs. In fact, the synthetic TLR7/8 agonist IMQ is commonly used for the treatment of certain skin tumors. However, the design of antitumor vaccines based on pDCs can represent an improved opportunity to boost and direct tumor-specific immune responses. Until now, pDCs from two different sources have been used for developing pDC-based vaccines that have been tested in phase I/II clinical trials: autologous pDCs from peripheral blood and allogeneic pDC cell lines. Although to a limited extent, both vaccine platforms have proven to be well tolerated, without evidence of severe toxicity, and are capable of inducing TAA-specific T cells responses. Interestingly, Tel et al. reported improved PFS and OS in melanoma patients who received the pDC vaccine compared to control patients receiving standard dacarbazine chemotherapy. At the same time, Charles et al. observed high levels of PD-1 in anti-vaccine T cells together with the expression of PD-L1 in cells from the TME, revealing a potential challenge of pDC-based vaccines in immunosuppressive TMEs. New pDC-based vaccination strategies could overcome these limitations and improve the clinical potential of pDCs. From this perspective, the combination of pDCs with immune checkpoint inhibitors and/or other adoptive cell therapies is an attractive alternative.

## Figures and Tables

**Table 1:** Clinical trials evaluating adoptive transfer of pDCs as a cell therapy platform for anti-tumor vaccine development.

Clinical Trial Identifier	Recruitment Status	Principal Investigator	Institution	Condition	Phase	Number of Participants	Therapeutic Product	Dose	Toxicity	Immune Response Observed	Clinical Outcome	References
NCT01690377	Completed	C J A Punt	Radboud University Medical Center	Metastatic melanoma	phase I	15	Natural circulating pDCs loaded with peptides derived from melanoma TAAs	0.3–3 × 10^6^ pDCs/injection	Grade 1 flu-like symptoms	Upregulation of CD80, CD83, CD86, MHC class I, and class II in activated pDCs Migration of activated pDCs in vivo Tumor-specific CD4+ and CD8+ T-cell responses	Median PFS: 4 months Median OS: 22 months	Tel, Aarntzen et al., 2013 [29]
		C G Figdor										
NCT01863108	Completed	J Plumas	Grenoble University Hospital	Metastatic stage IV melanoma	phase I	9	GeniusVac-Mel4: allogeneic PDC line loaded with four melanoma TAAs	4–60 × 10^6^ pDCs/injection	General disorders. Administration site events. Other adverse events (i.e., nausea, abdominal pain, and decreased appetite). Grade 3 adverse events (pain and lymphadenitis).	Antigen-specific T cells Recruitment of anti-vaccine T cells into the tumor bed	Stable disease for 16 to 48 weeks in 4 patients	Charles, Chaperot et al., 2020 [31]
		J Charles										
NCT02692976	Completed	W R Gerritsen	Radboud University Nijmegen Medical Centre	Prostatic Neoplasms	phase IIa	21	cDC2, pDCs or a combination of both loaded with three prostate TAAs	cDC2 vaccinations: 2–5 × 10^6^ cells per injection pDC vaccinations: 1–3 × 10^6^ cells Combined cDC2 and pDC vaccinations: 3–8 × 10^6^ cells	Grade 1–2 toxicity (flu-like symptoms, fatigue, upper respiratory infections, injection site reactions, etc.)	Antigen-specific T cells with no significant differences between treatments IFN-γ production	Median PFS for all patients: 9.5 months Median OS: not reached	Westdorp, Creemers et al., 2019 [30]
		F Witjes										
		J de Vries										
NCT04212377	Completed	J de Vries	Radboud University Medical Center	Metastatic Endometrial Cancer	phase II	8	cDC2 and pDCs loaded with TAAs					
NCT03970746	Recruiting	J Vansteenkiste	PDC*line Pharma	Non-small-cell lung cancer	phase I/II		Allogeneic PDC line loaded with TAAs					

## Data Availability

Not applicable.

## Abbreviations

APC	Antigen-presenting cell
BM	Bone marrow
BMT	Bone marrow transplantation
BST2	Bone marrow stromal antigen 2
CBT	Cord blood transplantation
cDCs	Conventional DCs
CLR	C-type lectin-like receptors
CRPC	Castration-resistant prostate cancer
CTLs	CD8+ cytotoxic T lymphocytes
CXCL	C-X-C motif chemokine ligands
CXCR3	C-X-C motif chemokine receptor 3
DCs	Dendritic cells
Flt3L	Fms-like tyrosine kinase 3-ligand
GM-CSF	Granulocyte-macrophage colony stimulating factor
HNSCC	Head and neck squamous cell carcinomas
HSPCs	Hematopoietic stem and progenitor cells
IDO	Immunoregulatory enzyme indoleamine 2,3-dioxygenase
IFN-I	Type I Interferon
IFNR	IFN receptor
IL-3	Interleukin 3
ILT7	Immunoglobulin-like cell transcript 7
IMQ	Imiquimod
IRF	Interferon Regulatory Factor
MHC	Major histocompatibility complex
moDCs	Monocyte-derived dendritic cells
NK	Natural killer
OS	Overall survival
PBMCs	Peripheral blood mononuclear cells
pDCs	Plasmacytoid DCs
PFS	Progression-free survival
PGE2	Prostaglandin E2
SLN	Sentinel lymph node
TAA	Tumor-associated antigens
TGF-β	Transforming growth factor- β
TILs	Tumor-infiltrating lymphocytes
TLR	Toll-like receptors
TME	Tumor microenvironment
TNF	Tumor necrosis factor
TRAIL-R2	TRAIL receptor-2
Treg	Regulatory T cells
